# Risk of Mortality in Association with Pregnancy in Women Following Motor Vehicle Crashes: A Systematic Review and Meta-Analysis

**DOI:** 10.3390/ijerph19020911

**Published:** 2022-01-14

**Authors:** Ya-Hui Chang, Ya-Yun Cheng, Wen-Hsuan Hou, Yu-Wen Chien, Chiung-Hsin Chang, Ping-Ling Chen, Tsung-Hsueh Lu, Lucia Yovita Hendrati, Chung-Yi Li, Ning-Ping Foo

**Affiliations:** 1 Department of Public Health, College of Medicine, National Cheng Kung University, Tainan 70101, Taiwan; yhccat@gmail.com (Y.-H.C.); yuwen32@gmail.com (Y.-W.C.); robertlu@mail.ncku.edu.tw (T.-H.L.); cyli99@mail.ncku.edu.tw (C.-Y.L.); 2 Department of Environmental and Occupational Health, National Cheng Kung University, Tainan 70101, Taiwan; B507092063@tmu.edu.tw; 3 Department of Environmental Health, Harvard University T.H. Chan School of Public Health, Boston, MA 02115, USA; 4 Department of Physical Medicine and Rehabilitation, Taipei Medical University Hospital, Taipei 11031, Taiwan; houwh@tmu.edu.tw; 5 Master Program in Long-Term Care, College of Nursing, Taipei Medical University, Taipei 11031, Taiwan; 6 Department of Geriatrics and Gerontology, Taipei Medical University Hospital, Taipei 11031, Taiwan; 7 Graduate Institute of Clinical Medicine, College of Medicine, Taipei Medical University, Taipei 11031, Taiwan; 8 Center of Evidence-Based Medicine, Department of Education, Taipei Medical University Hospital, Taipei 11031, Taiwan; 9 Department of Obstetrics and Gynecology, National Cheng Kung University Hospital, College of Medicine, National Cheng Kung University, Tainan 70101, Taiwan; ahsin@mail.ncku.edu.tw; 10 Graduate Institute of Injury Prevention and Control, College of Public Health, Taipei Medical University, Taipei 11031, Taiwan; plchen@tmu.edu.tw; 11 Department of Epidemiology, Biostatistics, Population Studies and Health Promotion, Faculty of Public Health, University of Airlangga, Surabaya 60115, Indonesia; lucia-y-h@fkm.unair.ac.id; 12 Department of Public Health, College of Public Health, China Medical University, Taichung 40402, Taiwan; 13 Department of Healthcare Administration, College of Medical and Health Science, Asia University, Taichung 41354, Taiwan; 14 Department of Emergency Medicine, An Nan Hospital, China Medical University, Tainan 70965, Taiwan; 15 Graduate Institute of Medical Sciences, Chang Jung Christian University, Tainan 71101, Taiwan

**Keywords:** injury severity score, mortality, traffic accident, pregnancy

## Abstract

The aim of the study was to provide a systematic review and meta-analysis of studies examining the association between mortality risk and motor vehicle crashes (MVCs) in pregnant women compared with nonpregnant women. We used relevant MeSH terms to identify epidemiological studies of mortality risk in relation to MVCs from PubMed, Embase, and MEDLINE databases. The Newcastle–Ottawa Scale (NOS) was used for quality assessment. For comparison of mortality from MVCs between pregnant and nonpregnant women, the pooled odds ratios (OR) with 95% confidence intervals (CI) were calculated using a random effects model. The eight studies selected met all inclusion criteria. These studies included 14,120 injured victims who were pregnant at the time of the incident and 207,935 victims who were not pregnant. Compared with nonpregnant women, pregnant women had a moderate but insignificant decrease in mortality risk (pooled OR = 0.68, 95% CI = 0.38–1.22, I^2^ = 88.71%). Subgroup analysis revealed that the pooled OR significantly increased at 1.64 (95% CI = 1.16–2.33, I^2^ < 0.01%) for two studies with a similar difference in the mean injury severity score (ISS) between pregnant and nonpregnant women. Future studies should further explore the risk factors associated with MVCs in pregnant women to reduce maternal mortality.

## 1. Introduction

Pregnancy is a common condition that increases the risk of severe injuries and poor outcomes, including maternal and fetal morbidity and mortality, following trauma [1]. Pregnant women who experience trauma are at increased health risk because they tend to suffer complications caused by an increase in soft-tissue edema and fluid response, and interpreting their vital signs is difficult due to altered hemodynamics. Surgical interventions may also be impeded because of their altered anatomy. These factors make trauma during pregnancy challenging to treat, and they contribute to poor adverse outcomes [2].

Motor vehicle crashes (MVCs) account for the largest portion of reported trauma during pregnancy [1]. Involvement in MVCs during pregnancy might lead to preterm labor, placental abruption, and fetal demise [3,4]. Moreover, excess maternal and fetal mortality can be attributed to various injury-related mechanisms during the crash. Even minor injuries may still cause severe adverse pregnancy outcomes because of delayed recognition of pregnancy(especially involving injuries suffered during the first trimester), maternal morbid obesity, and critically injured pregnant women. As a precaution, a previous study suggested that all women of reproductive age should be considered pregnant until proven otherwise [2].

Although pregnancy could complicate the outcomes after MVCs, existing evidence on whether pregnancy may lead to increased risk of mortality following MVCs remains inconclusive [5,6,7,8,9,10,11]. Some studies have revealed that the better survival rates after trauma among pregnant women are largely attributable to their tendency to be more cautious and concerned with their babies’ safety, and thus they are more likely to seek medical attention than nonpregnant women [11,12]. To summarize the available information on this topic, we conducted this systematic review and meta-analysis to determine whether pregnant women experience an increased risk of mortality after MVCs.

## 2. Materials and Methods

This review was conducted following the guidelines of the Preferred Reporting Items for Systematic Review and Meta-analysis (PRISMA) statement [13], the checklist of which has been included in Supplementary Checklist S1 (PROSPERO Review Protocol and Registration, CRD42020188698).

### 2.1. Eligibility Criteria

The inclusion criteria were as follows: (1) studies examined the mortality of MVCs, and (2) they were conducted on both pregnant women and nonpregnant women. The exclusion criteria were as follows: (1) case reports, qualitative reports, comments, simulation studies, and reviews; and (2) studies did not report information relevant for the key clinical questions, i.e., studies that reported fatalities of pregnant women due to MVCs but did not provide information about death following MVCs or traumatic injury.

### 2.2. Search Strategy

A comprehensive, systematic, electronic literature search was conducted using MEDLINE, PubMed, and Embase, regardless of publication timeline, in May 2021. References from the relevant literature were then manually searched and used as a basis for finding more relevant studies. The medical subject headings (MeSH) search terms used were ((Injured AND traffic injury OR accidents OR traffic OR motor vehicle) AND (pregnant or obstetric AND women) AND (mortality OR dead OR survival)) (Supplementary Table S1).

### 2.3. Risk of Bias Assessment

The revised version of the Newcastle–Ottawa Scale (NOS) for cohort studies was used to assess the quality of the included studies [14]. NOS contains eight items within three domains (selection, comparability, and outcomes). Studies that were assigned four stars for selection, two stars for comparability, and three stars for ascertainment of the outcome, were regarded as having a low risk of bias. Studies with two or three stars for selection, one star for comparability, and two stars for outcome ascertainment, were considered to have a medium risk of bias. Any study with a score of one star for selection or outcome ascertainment, or zero for any of the three domains, was deemed to have a high risk of bias [15]. Studies that were included in the review were assigned scores ranging from 0 to 9. Accepted standards were followed in the evaluation process to ensure objectivity in study selection. Two authors (YH and YY) independently performed all the data abstraction and then identified studies for inclusion or exclusion on the basis of NOS scores. Any discrepancy between the reviewers’ judgments regarding the scoring of a study’s quality was resolved by consensus with a third author (CY).

### 2.4. Data Extraction

The studies were evaluated on the basis of all relevant information provided in each article. The criteria for study inclusion were based on the relevance of the study’s setting (hospitalized or population-based study), the participants (pregnant and nonpregnant women following MVCs), location (the country which conducted the study), study design (retrospective or cross-sectional design), and sample size (the number of participants). These components were evaluated by the authors and, after the preliminary screening, eight studies were selected for further analysis.

### 2.5. Data Analysis

We used the odds ratios (OR) as our primary outcome to estimate the magnitude of the effect of pregnancy on the associated risk of death after women are involved in traffic injury. Crude OR were calculated or the adjusted OR of adverse pregnancy outcomes in MVCs victims, with or without pregnancy, were captured for individual studies, both of which were pooled to obtain an overall estimate using random effects models, as heterogeneity between studies was anticipated. Although data were extracted from each original study, the likelihood that some residuals and unexplained factors may exist in each article was acknowledged. Therefore, we proposed that the random effects model was suitable for use under these circumstances. A weighted mean was computed and pooled using a random effects model. Heterogeneity between studies was assessed using the I^2^ test (0–40%: might not be important; 30–60%: may represent moderate heterogeneity; 50–90%: may represent substantial heterogeneity; and 75–100%: considerable heterogeneity) [16]. All data analyses were performed using the Comprehensive Meta-Analysis Software Version 2 (Biostat, Englewood, NJ, USA). Sensitivity analyses were conducted by removing the study with the largest effect size. Subgroup analyses were accomplished according to the injury severity score (ISS) pattern of the study participants. On the basis of these analyses, an inference was made regarding whether an estimate of mortality could be attributed to the severity levels of the injuries sustained by the participants in the included studies.

## 3. Results

### 3.1. Characteristics of Included Studies

Of the 297 articles, 251 were selected and reviewed, and eight observational studies proved eligible (Supplementary Figure S1). These eight studies evaluated and comparatively analyzed the association among retrospective cohort studies (Table 1). These studies included 14,120 pregnant injured victims and 207,935 nonpregnant victims. Most of the studies involved multiple centers and used nationwide data sources to compare pregnant women and nonpregnant women.

According to the mechanism of injury, two articles enrolled only injured individuals from MVCs. Six articles enrolled trauma patients, of them 36.4–85.1% were due to MVCs (Table 1). Four studies used the age-matched method between pregnant and nonpregnant women (Table 1). The participants’ ages ranged from 12 years to 51 years. Many potential confounders were considered by the selected studies. However, the confounders they analyzed were diverse. Injury severity score was the most common potential factor they assessed.

### 3.2. Risk of Bias

**Figure 1:**
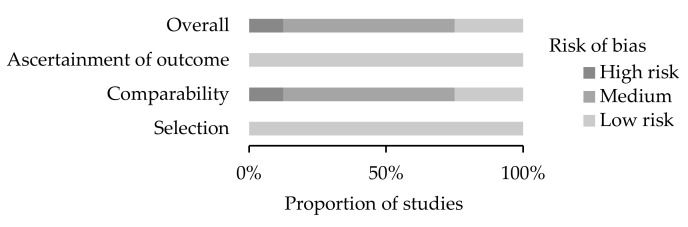
Quality assessment using the Newcastle–Ottawa Scale of studies included in the present systematic review (*n* = 8).

Seven studies were judged of medium/low overall risk of bias, and one study had a high overall risk of bias (Figure 1 and Supplementary Table S2). The main reason for downgrading was the lack of adequate comparability between groups, namely, limited adjustment for confounders between pregnant and nonpregnant women.

### 3.3. Overall Effects

Eight studies reported the number of deaths among pregnant and nonpregnant women. The number of participants, number of deaths, and ORs in the included studies are summarized in Table 2. In-hospital death records from Deshpande et al. [6] were captured to ensure that the definition of outcome was consistent among the included studies.

**Figure 2:**
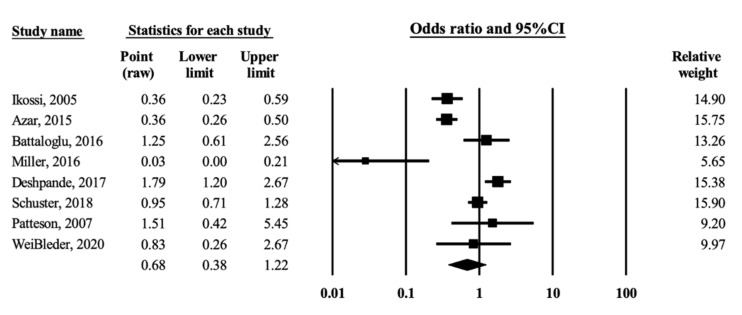
Forest plot of odds ratio for mortality from motor vehicle crashes in association with pregnancy [5,6,7,8,9,11,17,18]. The black squares represent the odds ratios of the individual studies and the horizontal lines are their 95% CI. The area of the black squares and diamond respectively reflects the weight each trial contributes and the overall effect from the meta-analysis.

In-hospital mortality was then used as the primary study endpoint. Among the eight studies, injured pregnant women were shown to have a moderate but insignificant association with a lower risk of in-hospital mortality than nonpregnant women after MVCs (OR = 0.68, 95% CI = 0.38–1.22; heterogeneity, Q = 62.03, df = 7, I^2^ = 88.71%, Figure 2).

### 3.4. Sensitivity Analysis

One outlier was identified. Sensitivity analysis was performed by removing the study with the largest effect size [8]. The results of the remaining seven studies were insignificant for lower risk of in-hospital mortality among pregnant women than among nonpregnant women (OR = 0.82, 95% CI = 0.47–1.44) but with moderate heterogeneity (Q = 51.72, df = 6, I^2^ = 88.40%).

### 3.5. Subgroup Analysis

Although four studies did not explicitly provide the mean ISS for the comparison groups [7,8,9,17], the values from two studies were nevertheless estimated by recalculating the mean through capturing the median of the classified ISS scale and the number of women injured by each ISS stratum [7,8]. Two studies did not provide any ISS information [9,17]. Differences in the mean ISS (ISSD) between pregnant and nonpregnant women were calculated, and the ISSD was categorized into ≤2 and >2. Among the women admitted to hospitals after an MVC, the pregnant women tended to have sustained less severe injures than their nonpregnant age-matched controls [9,17]. Therefore, those studies were considered to be in the ISSD > 2 group.

**Figure 3:**
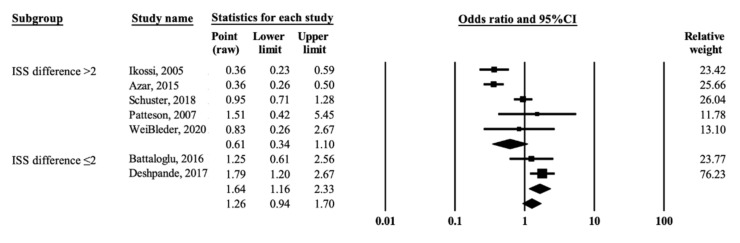
Forest plot of odds ratio for mortality from motor vehicle crashes in association with pregnancy according to differences in ISS between comparison groups [5,6,7,9,11,17,18]. The black squares represent the odds ratios of the individual studies and the horizontal lines are their 95% CI. The area of the black squares and diamond respectively reflects the weight each trial contributes and the overall effect from the meta-analysis.

Figure 3 shows the results of subgroup analyses which were performed by removing the outlier [8]. The pooled estimates from the two studies with ISSD ≤ 2 showed a significantly higher risk of mortality in pregnant women than in nonpregnant women (OR = 1.64, 95% CI = 1.16–2.33, *p* = 0.005) with little heterogeneity (I^2^ = 0%, *p* = 0.392). By comparison, the pregnant women tended to have a lower risk of mortality from MVCs than nonpregnant women in the five studies with ISSD > 2 (OR = 0.61, 95% CI = 0.34–1.10, *p* = 0.099) with obvious heterogeneity (I^2^ = 83.93%, *p* ≤ 0.001).

## 4. Discussion

According to the present systematic review, the issue of whether pregnancy increases the risk of mortality in victims involved in MVCs is controversial. This meta-analysis also found that, compared with nonpregnant women, pregnant women were found to have a moderate but insignificantly decreased mortality risk (pooled OR = 0.68, 95% CI = 0.38–1.22, I^2^ = 88.71%). To explore the sources of heterogeneity, we performed subgroup analyses according to differences in the ISS scale between pregnant and nonpregnant women. However, the pooled OR was significantly increased at 1.64 (95% CI = 1.16–2.33, I^2^ = 0.00%) for the studies with a similar difference in the mean ISS between pregnant and nonpregnant women. By contrast, the pooled OR tended to be reduced at 0.61 (95% CI = 0.34–1.10, I^2^ = 83.93%) in favor of pregnant women for the studies in which pregnant women had a lower mean ISS than their nonpregnant counterparts.

The mechanisms by which pregnancy might result in a low risk of mortality remain unclear. On the basis of our findings, we propose that when the inclusion criteria for maternal injury severity are considered, pregnant women have a higher risk of mortality than nonpregnant women due to pregnant women’s altered physiologic state, which in turn also affects their response to trauma [19,20,21]. Mothers obviously undergo physiological and anatomical changes during pregnancy, including increases in both plasma volume and cardiac output. These changes, combined with the shunting of blood away from the uteroplacental circulation after blood loss, may initially mask signs of hypovolemia [22]. Furthermore, pregnant women are at risk for aortocaval compression syndrome when in a supine position, and this may also confuse the interpretation of their vital signs [23]. These anatomical and physiological changes may affect the level of care extended to the injured trauma patient, thereby presenting a challenge to trauma surgeons. Moreover, these changes may explain the increase in mortality among women who sustain injury during pregnancy.

The inconsistency in the results of previous studies may be attributed to the potential for surveillance bias (also known as detection bias) [24]. Most studies reported that pregnant women are more likely to visit clinical institutions to confirm their baby’s safety after a crash than nonpregnant women, regardless of injury severity [11,25]. Furthermore, clinicians are prone to arrange admission for closer checkup or monitoring if a pregnant woman is hospitalized because of an MVC. Prior studies have demonstrated that nearly 60% of pregnant women were admitted to hospitals or emergency units after crashes, but only 34% of nonpregnant women were admitted [12,25]. Hence, well-monitored pregnant women had been observed to have a lesser injury severity than unmonitored nonpregnant women [11]. By contrast, among nonpregnant women, only those who sustained moderate injury severity were admitted to hospitals, leading to a misconception that nonpregnant women are associated with a higher risk of mortality after MVCs than pregnant women. This misconception was consistent with the present findings of a lower mortality risk in pregnant women than in nonpregnant women. However, a strong positive relationship between pregnancy and risk of mortality was observed after limiting the results to studies with differences in ISS of less than 2 between pregnant and nonpregnant women.

In general, the ISS scale is one of the most precise predictors of mortality in trauma patients. The ISS has been validated for predicting mortality after trauma in nonpregnant women [26]. However, Miller et al. [8] indicated that the ISS score might not be a useful predictive risk factor for pregnancy outcomes as they did not find a significant correlation between ISS and immediate adverse maternal–fetal pregnancy outcomes (*p*-value < 0.722). Schiff and Holt [3] also reiterated that the ISS is not accurate in predicting placental abruption in a study of hospitalized pregnant women following experiences of trauma. In fact, the relationship between the ISS and mortality risk in pregnant women is unclear due to the small number of maternal deaths reported in prior studies [3,12,27].

Apart from the potential confounding factor of the ISS, another possible confounder might have affected the results of previous studies. The possible dissimilarity in risk-taking behavior between pregnant and nonpregnant women, and its contribution to the low risk of mortality noted in previous studies cannot be discounted. Pregnant women may be more cautious in an effort to protect their babies. Previous studies show that pregnant women have better compliance with seatbelt use (66%) than nonpregnant women (50%) [11]. Therefore, pregnant women display behaviors that reduce risks, such as wearing a seat belt or being a passenger instead of the driver, and these behaviors may prevent pregnant women suffering a severe injury in a traumatic event. However, the possible risk reduction behaviors adopted by pregnant women were not considered in previous studies.

Selection bias is another possible source of bias in previous studies. Studies on the relationship between pregnancy and MVC-related mortality were conducted in various healthcare settings, including trauma centers or hospitals and clinic institutions. However, some injured victims might possibly have died at the scene and therefore, were not captured in data from clinical settings. Most medical institution-based studies enrolled trauma patients only when they visited or were transported to hospitals or trauma centers [5,7,8,9,11,17,18]. These patients’ conditions were likely to be categorized as moderate in relation to injury severity. A potential selection bias might arise if a study was based only on patients with MVC-related injuries seen in medical institutions [28]. The underrepresentation of out-of-hospital cardiac arrest (OCHA) cases might have also biased, at least to some extent, the results of previous studies. A population-based study design or patient enrollment that includes all pregnant women who experience MVCs may help reduce the potential selection bias (or survival bias) owing to the exclusion of OCHA cases or those cases where women did not seek clinical care after MVCs [6].

### Strengths and Limitations

We conducted this review by using a prospectively registered protocol and reported it in accordance with international standards [29]. To the best of our knowledge, this review is the first meta-analysis to examine the potential role of pregnancy in the associations between mortality and victims involved in MVCs. We used established tools to assess outcome reporting quality for the risk of bias [13,14].

The weaknesses of this systematic review are as follows. Firstly, the mean ISS was not available in two studies. Secondly, the adverse outcomes following MVCs, including mortality, can be affected by various factors, such as adherence to seat belt use [30], type of vehicle [31], and quality of medical care [32]. Unfortunately, very few studies considered these safety features in their analysis, thus limiting the interpretation of the present results on the relationship between pregnancy and mortality following MVCs. Thirdly, we found only a limited number of relevant studies that compared mortality after MVCs between pregnant and nonpregnant women. Furthermore, almost all of the included studies were conducted in the US, and the most recent one was published in 2020. Given that road conditions and safety driving features have improved in recent years, this metal-analysis was unable to provide the most up-to-date information. Fourthly, only two of the included studies specifically assessed injury severity following MVCs. The other six studies involved participants with trauma due to various causes. Nevertheless, most of the included studies evaluated MVCs as the major cause of trauma. Finally, we did not consider the initial (baseline) health conditions of the women in both groups in our analysis, primarily because none of the included studies mentioned the medical history of the study participants before the MVCs.

## 5. Conclusions

After risk stratification (i.e., ISS), this meta-analysis observed a moderate but positive association between pregnancy and mortality in women after MVCs. Some previous studies that compared mortality risk after MVCs between pregnant and nonpregnant women might have been affected by potential confounding factors, due to dissimilarities in injury severity and safety features. Additionally, prior studies based on clinical settings were subject to selection bias arising from the exclusion of OCHA cases, uninjured cases, and mildly injured cases who did not require medical care.

This review suggests a need for additional original research with improved methodology to provide better quantitative evidence concerning mortality risk in relation to pregnancy after MVCs. Understanding how pregnancy may be associated with the risk of death in the context of traffic incidents has the potential to lead to improved treatments, including both emergency medical and prenatal care.

## Figures and Tables

**Table 1:** Characteristics of studies included herein.

Author, Year of Publication, Country	Study Design	Study Duration	Data Source	Study Participants Injured in MVCs (%)		Age Range (Years)	Potential Confounders Considered
				Pregnant	Nonpregnant		
Ikossi et al. [11] 2005, US	Retrospective study	1994–2001	Multiple centers (130 trauma centers), National Trauma Data Bank	70.4	54.7	12–51	Mean age, mortality, mean ISS, mean LOS, SBP < 90, alcohol consumption, drug use, seatbelt use
Patteson et al. [17] 2007, US	Matched retrospective cohort study	1996–2004	Trauma registry at level I regional trauma center	85.1	NA	No restriction but age and time frame matched	Revised Trauma Score, admission to OR/ICU
Azar et al. [9] 2015, US	Matched retrospective cohort study	2003–2011	Multiple centers, national inpatient sample database	100.0	100.0	No restriction but age matched	Age, race, income, type of insurance, type of hospital, type of vehicle (motor vehicle, motorcycle, pedestrian),
Battaloglu et al. [7] 2016, UK	Retrospective review	2009–2014	National trauma registry	55.5	36.4	15–50 (with age matched)	ISS, AIS, blood transfusion
Miller et al. [8] 2016, Israel	Retrospective cohort study	2006–2013	National trauma registry	100.0	100.0	18–40	ISS
Deshpande et al. [6] 2017, US (Pennsylvania)	Retrospective cohort study	2005–2015	Pennsylvania Trauma Outcome Study	58.1	52.8	14–49	Age, race, Hispanic ethnicity, insurance type, comorbidities, GCS score, SBP, dead on arrival, ISS, injury mechanism, transferred to OR, intubated, required ICU admission, transfused, discharge category
Schuster et al. [5] 2018, US(Pennsylvania)	Matched retrospective cohort study	1999–2013	Pennsylvania Trauma System Foundation’s (PTSF) database	60.0	62.0	No restriction but age- and injury type- matched	Race, positive for drug screening, positive for alcohol screening, SBP, pulse, respiratory rate, GCS score, ISS, protective device, intubation, number of hospital days, surgical procedure performed
Weißleder et al. [18] 2020, Germany, Austria, and Switzerland	Retrospective data	2016–2018	Multiple centers Trauma Registry TR-DGU	60.0	47.0	16–45	ISS, AIS, Pre-hospital measure: intubation, respiratory aids, infusion therapy, infusion volume (mL), catecholamine therapy, chest drain, analgesic sedation, tranexamic acid, imaging procedure, intensive care, invasive ventilation; ICU length of stay, ventilation duration, complications

Abbreviations: ICU, intensive care unit; OR, operation room; GCS, Glasgow Coma Scale score; AIS, Abbreviated Injury Scale; LOS, length of stay; SBP, systolic blood pressure; NA, not available; ISS, injury severity score; MVCs, motor vehicle crashes.

**Table 2:** Characteristics of studies included herein.

Study ID	Country	Number of Participants		Number of Deaths		Odds Ratio (95% CI)	Mean ISS		Difference in Mean ISS (ISSD) between Groups
		Pregnant	Nonpregnant	Pregnant	Nonpregnant		Pregnant	Nonpregnant	
Ikossi et al. [11]	US	1195	76,126	17	2893	0.37 (0.23–0.59) ^1^	6.1	9.7	3.6
Patteson et al. [17]	US	188	188	6	4	1.52 (0.42–5.46) ^1^	NA	NA	>2
Azar et al. [9]	US	5936	59,360	39	1127	0.36 (0.26–0.50) ^2^	NA	NA	>2
Battaloglu et al. [7]	UK	158	14,082	8	576	1.25 (0.61–2.56) ^1^	11.7 ^†^	11.5 ^†^	0.2
Miller et al. [8]	Israel	3794	3441	1	32	0.03 (0.00–0.21) ^1^	1.4 ^†^	6.9 ^†^	5.4
Deshpande et al. [6]	US, Pennsylvania	1148	43,608	22	790	1.79 (1.20–2.67) ^3^	9.0	10.9	1.9
Schuster et al. [5]	US, Pennsylvania	1599	7995	54	284	0.95 (0.71–1.28) ^1^	5	9	4
Weißleder et al. [18]	Germany, Austria and Switzerland	102	3135	3	110	0.83 (0.26–2.67) ^1^	10.7	14.3	3.6
Total		14,120	207,935						

^†^ Mean ISS scale was estimated by each stratum of median of ISS and number of injured women. ^1^ Unadjusted odds ratio. ^2^ Adjusted for age, race, income, type of insurance, and type of hospital. ^3^ Adjusted for age, race/ethnicity, insurance type, Glasgow Coma Scale, injury severity score, and type of trauma.

## Data Availability

Not applicable.

## Supplementary Materials

The following are available online at https://www.mdpi.com/article/10.3390/ijerph19020911/s1, Checklist S1: PRISMA Checklist of the study, Figure S1: PRISMA flow chart describing the inclusion and exclusion processes, Table S1: Searching strategy, Table S2: Quality score of the included studies.
